# Back to the Basics: Response of Alopecia Areata Universalis to Intravenous High-Dose Pulse Corticosteroid Therapy

**DOI:** 10.5826/dpc.1104a60

**Published:** 2021-10-01

**Authors:** Kerasia-Maria Plachouri, Chrysa Oikonomou, Eleftheria Vryzaki, Sophia Georgiou

**Affiliations:** 1 Department of Dermatology, University General Hospital of Patras, Patras, Greece

**Keywords:** alopecia areata universalis, intravenous, pulse corticosteroids, remission

## Introduction

Alopecia areata is an autoimmune disorder of the hair follicles, with various subtypes, such as alopecia focalis, alopecia totalis, alopecia universalis (AU) and alopecia with ophiasis pattern [1]. AU refers to a complete loss of scalp and body hair, and it is considered to be associated with an unfavorable prognosis, both in terms of poor response to most treatments, as well as in terms of discouraging spontaneous remission rates, documented to be lower than 10% [1]. Despite numerous therapeutic attempts including with modern agents, such as biologics and JAK-inhibitors, everyday practice continues to rely frequently on the use of topical or systemic corticosteroids, with varying treatment outcomes [1–2]. Here we present the case of a patient with AU that showed an almost complete response to pulse treatment with intravenous methylprednisolone.

## Case Presentation

**Figure 1:**
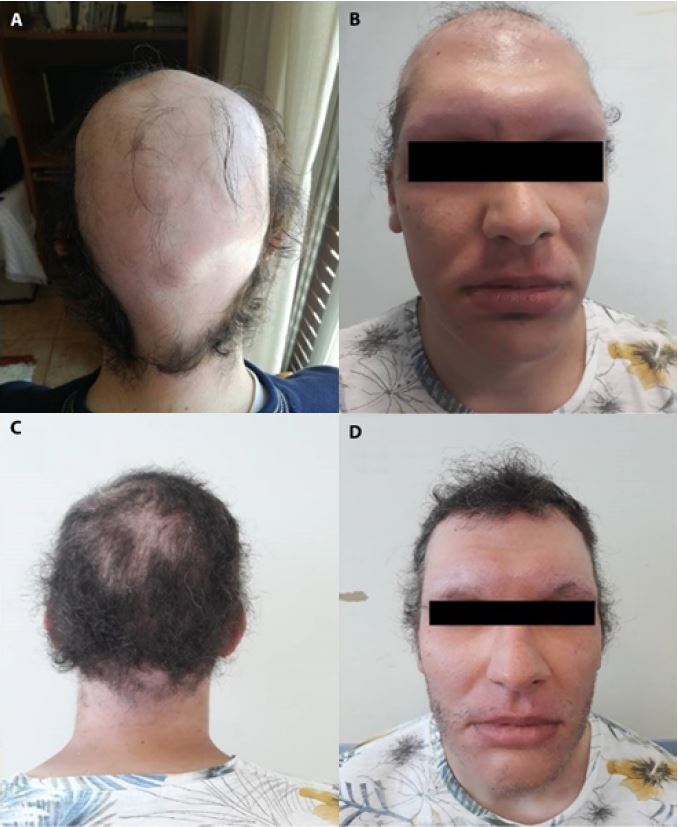
(A) Almost complete scalp hair loss. (B) Complete loss of facial hair. (C) Sustained hair regrowth in the scalp region during the 10-month follow-up visit after the cessation of the pulse methylprednisolone therapy. (D) Sustained hair regrowth on face area during the 10-month follow-up visit after the cessation of pulse methylprednisolone therapy.

A 34-year-old male patient presented to our dermatology department due to multiple hair loss patches on the scalp, eyebrows, and beard. Hair loss patches first appeared approximately 7 months prior to the referral. Other than elevated serum IgEs (1650 IU/ml), no other blood abnormalities could be detected. Due to progressive hair loss, despite a topical combination therapy with clobetasol propionate ointment 0.05% and minoxidil solution 5%, we opted for an intravenous corticosteroid pulse therapy (750 mg methylprednisolone in 250 ml dextrose over 3 consecutive days, every 4 weeks, for a period of 6 months), overlapped with the aforementioned ongoing topical regimen. Five months after therapy initiation the patient gradually lost almost all scalp and body hair (Figure 1, A and B). However, evidence of substantial hair regrowth was documented during the last methylprednisolone cycle, firstly on the scalp region and then, on the rest of face and body areas. After the cessation of the intravenous pulse therapy, the patient continued to use irregularly mometasone furoate solution 0.1%, as well as minoxidile solution 5% on the scalp region, in order to maintain hair regrowth. In the 10-month follow-up, significant hair regrowth was sustained in all previously affected areas of the head and body (Figure 1, C and D). Interestingly, the patient reported an alteration of the shape and texture of the newly regrown hair on the scalp region following the intravenous steroid treatment, from straight to curly.

## Conclusions

AU constitutes a therapeutic challenge for the physician and a significant psychological burden for the affected individual [1–2], due to the lack of established on-label treatments. In our case, hair regrowth during the course of the pulse therapy suggests a treatment response, rather than a spontaneous remission. The intravenous corticosteroid pulse therapy not only offers the advantage of minimizing steroid-associated side effects, but it is also linked with lower relapse rates for the patients who actually showed a response to treatment [2].
